# Spontaneous ethics in nurses’ willingness to work during a pandemic

**DOI:** 10.1177/09697330221085768

**Published:** 2022-05-13

**Authors:** Anna Slettmyr, Anna Schandl, Susanne Andermo, Maria Arman

**Affiliations:** Department of Neurobiology, Care Science and Society, 27106 Karolinska Institutet, Huddinge, Stockholm, Sweden; Department of Anaesthesia and Intensive Care, Södersjukhuset, Stockholm, Sweden; Department of Clinical Science and Education, Södersjukhuset, 27106 Karolinska Institute, Stockholm, Sweden; Department of Molecular medicine and Surgery, 27106 Karolinska Institute, Stockholm, Sweden; Department of Neurobiology, Care Sciences and Society, 27106 Karolinska Institute, Huddinge, Stockholm, Sweden

**Keywords:** Caring ethics, ethical demand, qualitative, hermeneutics, phenomonological, ontology, Løgstrup, Martinsen

## Abstract

**Background:** In modern healthcare, the role of solidarity, altruism and the natural response to moral challenges in life-threatening situations is still rather unexplored. The COVID-19 pandemic provided an opportunity to obtain a deeper understanding of nurses’ willingness to care for patients during crisis.

**Objective:** To elucidate clinical expressions of ontological situational ethics through nurses’ willingness to work during a pandemic.

**Research design, participants and context:** A qualitative study with an interpretive design was applied. Twenty nurses who worked in intensive care unit at two Swedish hospitals during the first, second, and third waves of the COVID-19 pandemic were interviewed. The analysis was interpretative and applied a theoretical ethics perspective.

**Ethical considerations:** The study was approved by the Swedish Ethical Review Authority and informed consent was obtained from all participants.

**Findings:** From a philosophical perspective, the nurses expressed sovereign life expressions of mercy and compassion, which arose spontaneously in response to seeing vulnerable fellow humans. They referenced ‘‘the nurse inside me’’ and their choice of profession as motives to provide care. Ontological situational ethics in culture and norms were noted in the constructs of competence, responsibility, solidarity with colleagues and organization; and interest and learning were driving forces. Ethical demand was evident when nurses expressed ideas of meaningfulness in helping their fellow humans; but themes of ambiguity, exhaustion and unwillingness were also present.

**Conclusions:** The nurses showed a high willingness to care for patients during a crisis. Responding to the ethical demand and to care for vulnerable human beings while risking their own health and lives could be interpreted as an inter-human vocation. These spontaneous altruistic actions saved the lives of many patients during the pandemic and need to be understood and supported

## Introduction

Humanity is vulnerable but also responsible and caring, an axiomatic truth which we all experienced during the multiple crises of the COVID-19 pandemic. People develop new behaviours and attitudes in life-threatening crises which are different from how they act and think in everyday life. Their understanding of life can thereby be turned upside down.^1^ Fundamental existential conditions, such as mortality, vulnerability and considerations as to the meaning of life become more prominent.^2^ What role solidarity and altruism have in modern society and whether they carry healthcare professions in times of crises is a question in professional ethics.

### Background

Our research group has previously studied expressions of altruism from an ethical perspective and reflections on responsibility for patient care in relation to the risk of one’s own health.^3^ In 2014, we conducted an interview-based study on altruism in nursing which showed ambiguity and hesitation as to whether they felt they would respond to the ethical demand of patients in the case of, for example, a pandemic.^3^ The COVID-19 challenged this ambivalence. In 2020, the world’s healthcare system was faced with a situation that required enormous efforts by frontline healthcare workers.^4^ Intensive care and affiliate specialities were particularly challenged as the disease caused severe respiratory problems. Intensive care unit (ICU) nurses were frontline workers in the care of these patients and nurses’ willingness to work hard during the pandemic was challenged.^5^ There is some evidence that nurses’ dedication, personal sacrifices and collegiality increased during the initial phase of the pandemic.^5,6^ Among graduating nurses, 82% expressed willingness to voluntarily care for these patients.^7^ In China, willingness among frontline nurses was 96%^8^; while in Australia, only 60% were willing to care for COVID-19 patients.^9^ These inconsistencies in an underexplored research area indicate a need for a more qualitative, deepened understanding of the topic. Many ethical questions will remain in the aftermath of this pandemic.^10–12^ Among them are ethics surrounding the traumatic experiences and stress responses among nurses.^13^ Wang et al. found low levels of stress among healthcare workers in China during the pandemic and concluded that this was a sign of devotion and altruism.^14^ Hossain et al. feared that nurses will suffer from moral injuries and long-term psychological wounds and emphasized the need to provide advice, self-help and support.^15^

Few qualitative studies have been published about natural responses to moral challenges in life-threatening situations, such as those experienced during the pandemic. So far, no study has related these experiences to philosophy of life issues. In this interpretive article, we explore a deeper understanding of ICU nurses’ willingness to care for patients during periods of crisis, specifically the ongoing pandemic. The study draws on a philosophical ethics perspective of ontological situational ethics.^16,17^ The purpose of this study was to elucidate clinical expressions of ontological situational ethics through nurses’ willingness to work during a pandemic.

## Methods

### Research design

The study was designed using a qualitative approach, including a theoretical interpretation of applied spontaneous ethics. The analysis followed a phenomenological hermeneutical method,^18^ with an interpretive attempt at merged horizons between the new data and the preunderstanding.

### Theoretical departure

As questions pertaining to our data focused on nurses’ willingness to work in a pandemic, the ethical demand was ‘close’, as articulated by Løgstrup, and further concretised by Martinsen, a nurse–researcher. The theoretical perspective was therefore based on Martinsen’s^16,19^ and Løgstrup’s^17,20^ philosophy about the ethical triad that consist of 1) sovereign life expressions, 2) the ethical demand and 3) interaction with relative cultural norms. In line with Løgstrup’s phenomenology, there are ontological phenomenon in human relationships that are not created by man, and which can manifest in spontaneous impressions and actions. Sovereign life expressions like trust; compassion and mercy; the ethical demand to care for others and relative culture-bearing norms constitute a conceptual map for ontological situational ethics,^16,19^ briefly illustrated in Table 1. Our purpose was to explore whether situational ethics can be better understood through nurses’ stories about their behaviour during the pandemic.

**Table 1.** Triad of ontological situational ethics.

Triad of ontological situational ethics		
Sovereign life expressions: mercy and compassion	The ethical demand to care for others	Relative cultural-bearing norms
Demonstrated as pre-reflective readiness for actions after seeing vulnerable fellow human beings	Appears when encountering fellow humans and experiencing their vulnerability	Involves a range of patterns and habits which can facilitate or hinder ethical behaviours

### Participants and research context

Data collection derived from individual interviews designed with open questions and conversational in nature and were conducted by experienced nurses in collaboration with researchers. Interviews were carried out with 20 frontline nurses who were on duty in the intensive care unit (ICU), taking care of patients with COVID-19, during the first, second and/or third wave of the pandemic in 2020 and 2021. Fifteen women (aged 27–64) and five men (aged 30–56), all with specialist training in intensive and/or anaesthesia nursing care joined the study (out of 27 total invited). The interviews lasted between 30 and 100 minutes and were conducted in a private room at the hospital (*n* = 15), on telephone (*n* = 1) or as digital web-interview (*n* = 4). The interviews were done with openness to the phenomenon of willingness to work during a crisis like a pandemic. Nurses who chose not to participate in clinical work were excluded from this study as the focus of this research project was willingness to perform clinical work during the pandemic.

In the Stockholm region, crisis agreements (for work shift duration and financial compensation) were activated for nurses and other healthcare workers employed in ICUs. Depending on the hospital, there were differing degrees of flexibility and voluntariness built into the system. Some nurses were not given a choice as to whether they would work longer shifts (12.5 hours instead of the normal 8-hour shifts and a weekly obligation to work roughly 60 hours or more instead of 38), while others could choose whether they wished to be included in the crisis agreement. The nurses also had to engage in the regular care of other ICU patients, in parallel with COVID-19 patient treatment.

### Analysis

The analysis followed the methods outlined by Lindseth and Norberg,^18^ where a combination of phenomenology and hermeneutics was used. The analysis was carried out in three steps; in the first step, entire transcripts were read to allow for an intuitive primary understanding. The entire research team (four members) read each interview separately. These readings were followed by a discussion, and notes were taken. Initial assumptions and questions emerged in relation to the reading of data, comprising a *naïve understanding*. This was the starting point for structural thematic analysis. During the second step, the *thematic analysis*, the nurses’ narratives and experiences were viewed as openly as possible by the researchers, keeping the preunderstanding in brackets while condensing the content. Meaning units were identified and sorted, and preliminary themes were underlined. The themes were then reflected on in relation to data and the naïve understanding. In the third step of analysis, *interpreted comprehensive understanding,* consistent with a fusion of horizons, took place in which all the different parts were merged. The data, the theoretical understanding of spontaneous ethics and the clinical thematic pictures emerging from the data were then interpreted in a process resulting in a new whole.

### Ethical considerations

The study was approved by the Swedish Ethical Review Authority (Diarie number: 2020–02699) and conforms to the principles described in the Declaration of Helsinki. The research project was planned in collaboration with the ICUs and the researchers. Participating nurses received verbal and written information and gave informed consent. Psychological support was accessible at each clinic in case it was needed. Participating nurses expressed satisfaction with being given the opportunity to share their story. Confidentiality and participant anonymity are guaranteed for both the transcriptions of the interviews and the quotations used in this analysis.

## Findings

### Naïve understanding

The first impression when listening to and reading interviews was how serious and shocking nurses perceived the clinical situation to be during the pandemic. Stories and descriptions were never-ending, as was the need to share these experiences with a listener. The nurses experienced they had participated in a unique and historic situation that they never imagined, and which they referred to as a ‘war zone*’*. Nurses were excited about being involved in the management of patients who suffered from a completely new disease and a health care situation that they had never experienced before. This enthusiasm to be part of a unique situation contributed to their willingness to work and care for those patients. Because of the magnitude of the crises, they pushed their limits to the maximum and shifted their boundaries continuously and performed sacrifices in solidarity with both patients and colleagues. In reply to the question about their willingness to accept long and hard workdays during a chaotic situation, and the risk of becoming infected, the first impression was that the nurses were surprisingly unaware of the motives behind their choices. The expression ‘naturally*’* preceded most replies, which indicates a self-evident approach. With the need for their specific competencies and skills came a sense of moral responsibility as well as a sense of expanded solidarity. The nurses compared their profession with others linked to social responsibility, like fire brigades or police, and the duty to perform their profession when society needed them to do so. Not fulfilling this duty was an unthinkable alternative and a choice they expressed they could not have lived with. Nurses reflected on what their choice of profession meant and identified as people who wanted to help others. They expressed pride and satisfaction, but also present were feelings of fatigue, complaints, reluctance and ambivalence towards working in a pandemic. Not having enough strength when needed generated feelings of despair and deficiency.

### Empirical thematic structural analysis

#### Driven by the situation, work efforts became self-evident

The situation in and of itself motivated the nurses’ intense work efforts in the ICU. They could not deny the severity of the situation. The news coverage was non-stop, and nurses realized they were needed in the ICU. They emphasized the obvious need in the request and compared their own efforts with those of others in situations of crisis where people spontaneously helped each other. The severity of the situation was even perceived as an internal ‘order’. One nurse described how they prepared for the oncoming increase in patient flow; ‘*Somehow, in February and March, we were like soldiers standing to attention’*. The situation made an extraordinary demand ordinary; one in which they simply wanted to help their fellow human beings. The natural, simple and evident choice to stand up for others was described as ‘*If I can help, I want to’*. The choice was also expressed as inevitable, despite the difficult situation and the risk of being infected by the virus. The nurses realized that this was an extraordinary situation where chaos reigned and hard work was required, but they still did not hesitate to give of themselves and share their knowledge with others, often in ways in which they had no previous experience. ‘*Who else?’* expressed a recurrent feeling that guided nurses’ willingness to work, particularly during the first wave, which meant that they could not opt out of shifts**.** Fatigue and long work shifts were endured as they were motivated by the idea that there was no one else who could perform their tasks. There were several descriptions regarding how they were out of energy, but the urgency of the situation demanded they continue working. When boundaries were pushed by the increasing amount of severely ill patients, contradictions between ‘a growing need’ and ‘strength to endure’ were described. The nurses worked more and harder than they actually were capable of, but at the same time, they expressed that it was not unreasonable based on the crisis situation that prevailed. To stay in the ICU and save lives was experienced as an obligation that had to be prioritized before everyday home life.

It felt as if I had to do to it, because we were in a catastrophic situation, and I had more important things to do than to be involved in the soccer school. Such trivial things. Here I am saving lives.

#### Interest and learning as driving forces

In the beginning, the new situation served as incentives for hard work and evoked a willingness to stand up for others. The abruptness of the pandemic challenged everything but also inspired an interest in the event. The nurses’ competence and creativity were challenged, and they went beyond normal routines. This gave them the opportunity to test the limits of their knowledge in a situation comparable to accidents or to war, which was perceived, in many ways, as a positive challenge. One nurse stated, ‘*In some way, I would not have wanted to be without this experience’.* In hindsight, to be part of a unique historical situation, together with the attention ICU care attracted, created feelings of self-respect and pride in having participated in the pandemic response, and this contributed to feelings that they had won more than they lost. However, the experiences were like a double-edged sword, exciting and educational, but also demanding and exhausting.

#### Solidarity with colleagues and the organization

The pandemic created pressure from a growing population of severely ill people to an extent the nurses had never experienced before. The ICU had to be rapidly reorganized. Group affiliation and collegiality increased their energy and a desire to work, and collaboration was highly appreciated, in their experience that there was no one else who could do the tasks. However, inside the group, the nurses felt that an indirect pressure arose, with a common focus on taking on increasingly demanding tasks. The pressure was not only experienced as a negative, but also contained supportive components. The expressions ‘*Let’s do this’* or ‘*Just do it’* showed that life-saving procedures surpassed every other need. They did not want to abandon their hardworking colleagues and solidarity was highly valued. One of the nurses expressed that they primarily worked to support each other and regarded collegiality as superior to any other driving forces. Another nurse found team coherence of great importance for the endurance; ‘*We have carried each other through this pandemic’*.

#### With competence comes responsibility

Knowledge and competence were essential components in the choice to voluntarily enter pandemic care. The competence of an ICU nurse was suddenly the highest priority of a whole society, which increased their sense of responsibility. Since competence and need were so closely intertwined, nurses felt they had no other choice but to submit to the task at hand, and they began to see their competence as something that was not only vital but also as something that contained within it a sense of duty. Their internal moral compass required that they succumbed to the work. There were no other options. The realization that they possessed the necessary competence and were the only ones that could do what needed doing came with a responsibility to fellow humans and to society at large. The pandemic made nurses realize that it was their turn to stand up for society. ICU nurses became indispensable.

In a war situation, it is expected that armed forces will defend the country. It is similar to the pandemic; everyone expects that healthcare workers will step up and solve the situation.

The importance of this professional experience was captured by a nurse who stated, ‘*I have trained for this all my life*’.

#### The choice of profession as a motive

The personal choice to become a nurse, to be able to help others, indicated that they were ‘that kind of people’, altruistic by personality. The spontaneous desire to stand up for others in need evoked reflections and it was emphasized that they were individuals who stood up for others. ‘*We have it in us’*, was said among the nurses, who expressed themselves as a collective. Others stated that it was their conscience or their moral code that made them stand up for the patients during the pandemic. They could not have lived with themselves if they had not. ‘*It would be too painful not to act’*, was one nurse’s motivation, and another stated; ‘*The nurse in me says that we must help each other and… join in’*. The motive behind becoming a nurse was both evoked and received new significance in the light of the pandemic. Several nurses restated their basic, intrinsic values, which had once motivated their choice of career. They emphasized that particularly at the beginning of their careers they were motivated by a desire to help others. This was a foundation and attitude that recurred during the pandemic that they wanted to maintain in the future. There was an awareness of nursing as a profession that asks its practitioners to stand up and take risks for others. One informant expressed that she had considered this when she applied to nursing school and concluded that she was willing to put herself second, behind others who needed her care. Several nurses mentioned that they previously had considered working for foreign aid organizations, which they thought required equal sacrifices to those experienced working during the pandemic.

#### The meaningfulness in helping a vulnerable human being

The pandemic was perceived as an eye opener, reminding nurses that the basic conditions of life are fragile, and that human beings are vulnerable and at the mercy of others. Nurses realized that others were dependent upon them; they could not abandon those who were seriously ill. Patients were fragile and vulnerable and trusted nurses with their lives, which meant they were under an obligation to respond to the needs of their fellow humans. The encounter with severely ill patients, with their fragility and dependence, led to questions about life and its meaning for the nurses. They reflected on how small things became less important and how fragile their own lives were. A recurring thought was that the patient could have been me or someone I care about. One nurse stressed ‘*life is about give and take’*. The satisfaction of being able to ‘do good’ for others was noted, mostly where the concepts ‘natural’ or ‘self-evident’ occurred in the nurses’ narratives. The willingness to be involved in pandemic care was described as an emotional desire to, through your deeds, be of importance to another human being in a difficult situation. Further, it felt good, even enjoyable to be able to help others, to do good and meaningful things, but also to receive appreciation and acknowledgement for their deeds. Despite the severity of the situation, the meaningfulness of work assignments and the satisfaction in completing them led nurses to perceive the work as rewarding.

#### Ambiguity, exhaustion and unwillingness

For some nurses, however, the first reaction to updates about the pandemic on the news was one of pure fear; ‘*Must I, is there no one else who can go into the ICU?’* Initial ambivalence and anxiety increased when they realized that something extraordinary might be required. Some nurses expressed ambiguity about the situation. On one hand, nurses felt important and enjoyed being needed. On the other hand, the situation was extremely difficult, exhausting, and sad. All the suffering they witnessed and traumatic events they experienced led to reluctance to continue the work. They wondered whether they would have the strength to work during the crisis agreement again and hoped they would not be forced to do so. However, they found it difficult to deny patients help and assumed they would probably work again if needed. The work team could joke about the absurdity of the workload, but they still remained by the patient’s side. The severity of the situation and the necessity of their presence ensured that they never seriously debated giving up. ‘You are able to do more than you actually think’ was a recurrent theme, and one nurse said that she thought she would manage only 2 weeks on the crisis agreement but remained at her posting for 6 weeks during the first surge. Some nurses expressed how they almost worked themselves to death, but all the same, found a necessity in their endeavours. Due to the pandemic, several nurses became ill by infection or because of the work burden, while others resigned on their own initiative. Becoming ill by COVID-19 or other illnesses was described by some nurses as receiving a highly desired pause from work, an ‘emergency exit’. Exhaustion took its toll, and some colleagues suffered from posttraumatic stress. For some, this was where willingness ended. One nurse described that she wanted to work, but after weeks on the crisis agreement, the fatigue was so exhausting that she did not recognize herself, and she was unable to handle the situation. Others suffered from burn out and experienced how the situation exceeded their capabilities.

People are crying behind their protective masks, hyperventilating and having panic attacks. They hit the wall and must go on sick leave. I think this has gone too far.

### Comprehensive understanding and interpretation

The data and the thematic analysis illustrate how nurses think and act in situations where they are confronted with the important assignment of saving lives in a pandemic, in a chaotic situation and at the risk of infection. It is clear how mercy and compassion interact with the severity of the situation and the responsibility inherent in the commitment, which may explain why they choose to expose themselves to danger and to long work shifts during new and chaotic circumstances. The patients’ potentially fatal condition inspires feelings of mercy, and an ethical demand, when nurses express the idea that they are saving lives here and that it is not an ordinary job anymore. There is a paradox in that the nurses spontaneously sacrifice themselves to do good for patients but do not perceive their own actions as good. Their choices remain self-evident and show signs of an inter-human vocation to care for vulnerable fellow humans. Trust becomes visible when the nurse knows that the patient has no choice but to put his/her life in the hands of the nurses, and the nurse, in turn, experiences the vulnerability and fragility of life. When sovereign life expressions are activated in tandem, solidarity and common goals arise; compassion and patient vulnerability set the agenda. The pressure to perform and support the group is facilitated by spontaneous trust. In encountering vulnerable fellow humans, nurses rose to the ethical demand, citing the need to do so as self-evident, which could be interpreted as a vocation to do good for their fellow humans.

The themes presented in the structural analysis are inter-related and are outlined using interpretive assumptions related to the ethical triad in Table 2.

**Table 2.** Themes with quotations and the theoretical triad of ontological situational ethics.

Theme	Example of quotation	Ethical triad
Driven by the situation, work efforts became self-evident	Exactly, it was just to join in. There was nothing to consider, you did not refuse to go there because you wanted more information, it was all in the air, ‘this is serious’, so I did not question it	Sovereign life expressions of mercy and compassion arise spontaneously in sight of a vulnerable fellow human
Interest and learning as driving forces	But I thought it was almost a bit exciting, yes, I felt ‘now I get to test my limits’ a bit	Culture and norms – the learning process is beneficial for me as well as for my surroundings
Solidarity with colleagues and organization	My colleagues worked like dogs. That is probably why I also did it	Culture and norms – the situation called for professional fellowship
With competence comes responsibility	‘Morally speaking, there is no choice. It does not matter, you must [do it], if you have the knowledge, you also have the responsibility’	Culture and norms – the duty of being a part of a profession and an organization interact with an ethical demand
The choice of profession as a motive	I felt that rather quickly, it is probably the nurse inside you that makes you feel that we all need to contribute. So immediately when COVID-19 spread, I felt that I am ready (to participate)	Ontology in situation – being a fellow human being in a certain profession
The meaningfulness in helping a vulnerable human being	Anyway, ‘it could have been me’	The ethical demand to consider taking care of whomever you meet
Ambiguity, exhaustion, and unwillingness	Inside you, it is two-sided. On the one hand, it is exciting, it is an exceptional situation we are in…and at the same time, it is difficult, it is sad and it is exhausting	Hindrances and abandonment of the ethical demand introduce despair and guilt

If we look at the ethical triad according to Martinsen,^16,19^ which consists of sovereign life expressions, ethical demands and pre-existing cultures and norms, they all interact with each other. In ontological situational ethics, each situation is unique but also has unifying features, visible as nuanced coherence in our data. Ontology relates the basic conditions of life which means that we are concerned with each other and are constantly dependent on each other, which is also conceptualized as interdependency, as expressed by the nurses in the concept of ‘who else?’ in a situation where something must be done for another person, and the realization that they are the right person for this particular calling. In line with interdependence – a preconscious life expression of reciprocal dependence – the idea of responsibility emerges as something one does not choose but something that belongs to the pre-existing conditions of life. All nurses agreed on that standing up for others, when their knowledge was needed, was a matter of course. This invisibility in what defines and delimitates ‘willingness’ is the foundation of life expressions, which Martinsen describes as hidden, anonymous and overlooked while simultaneously supporting human relationships.^16^ These actions were carried out casually and with no ulterior motives, and nurses expressed the situation as strenuous but also exciting, since the nurses’ specific knowledge was needed at that moment in time. Sovereign life expressions including the phenomena of trust, compassion and mercy capture people, inspiring them to stand up for one another in crisis situations. Signs of the triad of situational ethics are actions done with ease and joy. However, anything else would be unthinkable. Nurses focused on life-saving procedures, said ‘let’s go’ and worked 12-hour shifts until further notice. Relative norms and cultures within the work situation slowly changed, and nurses followed and adapted to the new situation despite describing it as a war zone. An ethical demand implies a responsibility to comply with what the situation requires. It is not clearly stated but lies in the trust that has been placed with the nurses, and what comes their way. When the nurse says that she has trained for this all her life, it may demonstrate a strong activation of the ethical demand. Similarly, statements like ‘my competence was needed’, and ‘I have a responsibility because I have the knowledge’, are expressions of ontological spontaneous ethics. According to Martinsen,^16^ the ethical demand is one-sided and puts pressure on the recipient to respond to other’s vulnerability. This demand tells us what we owe another individual, with or against our will. Culture and norms play a part in this, and its inherent ‘ought to/should’ dynamic establishes ethics and induces the self-evident part of the experience.

## Discussion

Combining empirical data with ontological philosophy in this study show how life issues and the lived experience are woven into each context. Previously, we have shown how spontaneous expressions such as mercy, compassion and interdependency manifest themselves in crises,^1^ while we want to point out that both everyday life and life in a clinic are full of potential crises. Our lives as relational and interdependent individuals call us to care for, and to take care of, what is placed in our hands. Compassion as a sovereign life expression in nurses’ work in the ICU has been extremely challenging during the pandemic as it involves much suffering and many deaths occurring in a frustrating context. On the one hand, different driving forces like compassion – an inner moral duty to care for the life of the vulnerable human being that was put in the hands of the nurses – and other sovereign life expressions, gave the nurses the possibility to answer the ethical demand and act with ‘neighbourly love’ toward their fellow humans.^21^ On the other hand, compassion fatigue has been the result among some colleagues^22^ and is a crisis described as experiences of negative emotions of guilt, shame and anger which has consequences for the person as well as for the profession.^23^ The nurses who lacked the strength to bear the workload became sick or resigned from their jobs. Ontological situational ethics supports choice in actions, as long as they are spontaneous, particularly during the beginning of the pandemic. In the longer term, Løgstrup believes that reflections can go either way and spontaneous life expressions can be rejected,^17^ which is visible in the themes surrounding the constructs of ambiguity and unwillingness. It is important that the demands and relative norms of the organization can interact and support ontological situational ethics because if the ethical demand is difficult to maintain, nurses might resist it. ‘I betray myself when I fail [to fulfil] my responsibility for others’ well-being’ Martinsen writes.^16^ To be in haste and to lack time in a situation is compatible with ethics; but when norms lead to numbness towards the fellow human, feelings of shame, guilt and alienation arise. This is an important part of the ethical aspect of caring for patients during a pandemic to respect and understand.

In this study, the nurses prioritized the needs of their fellow humans before their own needs and pushed themselves beyond their limits, which can be interpreted as an expression of altruism as well as vocation. The understanding of and respect for such altruistic actions are of importance for supporting nurses’ vocational approach to care for vulnerable patients. When life expressions and context agree, ethical behaviour is facilitated. However, a failure to follow these demands may give rise to feelings of despair, as shown in this study. Martinsen believes that according to the ethical demand, cherishing life and nurturing the life that is placed in our hands is a basic human phenomenon.^21^ Without this expression of vocation from the nurses, the health care would not have been able to provide care to all these patients during the pandemic. The altruistic behaviour of nurses’ vocational approach to their duty has been of utmost importance during this crisis and must be confirmed and supported in case of future crises.

Compassion satisfaction among nurses can grow and increase in the context of pandemic care.^22^ During the pandemic, most nurses faced challenges and gained life experiences beyond anything they had hitherto experienced, leading to existential challenges and growth as well as personal fatigue. As the triad of ontological situational ethics was intertwined, the willingness of nurses to work remained intact and eventually the triad became harmonious.

### Strengths and limitations

Conducting in-depth individual interviews was helpful in gaining access to the nurse’s narratives regarding their willingness to care for patients during a pandemic. Some of the interviews were held with help of digital media or telephone, which could have negatively influenced the quality of the interviews and the interaction between the participant and the researcher. Due to the pandemic, there was an adaption to the use of digital media in society, which possibly made participants and researcher more comfortable with conducting in-depth interviews online. The interviews were held during a 10-month period, which could have affected the data, as the nurses’ experiences and attitude to work during a pandemic could have changed with time. On the other hand, it could be positive in gaining a broader understanding of the phenomena under study due to variations of experiences and attitudes during the pandemic. The participants who were included in this study were still working in the ICU, and nurses who were on sick leave or had chosen to finish their employment were left out. These nurses will be the focus of a future study where their experiences of not being able to care for patients during a pandemic will be explored.

## Conclusion

This study focused on questions surrounding nurses’ willingness to work in the ICU during an ongoing pandemic by adding a theoretical perspective. The paper puts the ethical triad in context with clinical evidence and may contribute to a deeper understanding of ontology and the meaning of situational ethics in nursing care. In line with hermeneutics, knowledge and understanding of phenomena lead to their natural application and provide a foundation for reflection. In this study, we point to the ontology of being human and the spontaneous life expressions of interdependency that arise in the encounter with fellow human being’s vulnerability and suffering. The nurses showed a high willingness to care for patients during a crisis. Responding to the ethical demand and to care for vulnerable human beings while risking their own health and lives could be interpreted as an inter-human vocation. These spontaneous altruistic actions saved the lives of many patients during the pandemic and need to be understood and supported.
